# Supplementary material for: Causal Relationship between Aging and Anorexia Nervosa: A White-Matter-Microstructure-Mediated Mendelian Randomization Analysis
Source: Biomedicines. 2024 Aug 16;12(8):1874. doi: 10.3390/biomedicines12081874 (PMC11351342; doi:10.3390/biomedicines12081874)
Supplement: Supplementary file 1 [file biomedicines-12-01874-s001.zip › Supplementary Figure/Figure S4/Singe SNP plot of MR analysis for TL to ubm-b-2102.pdf]

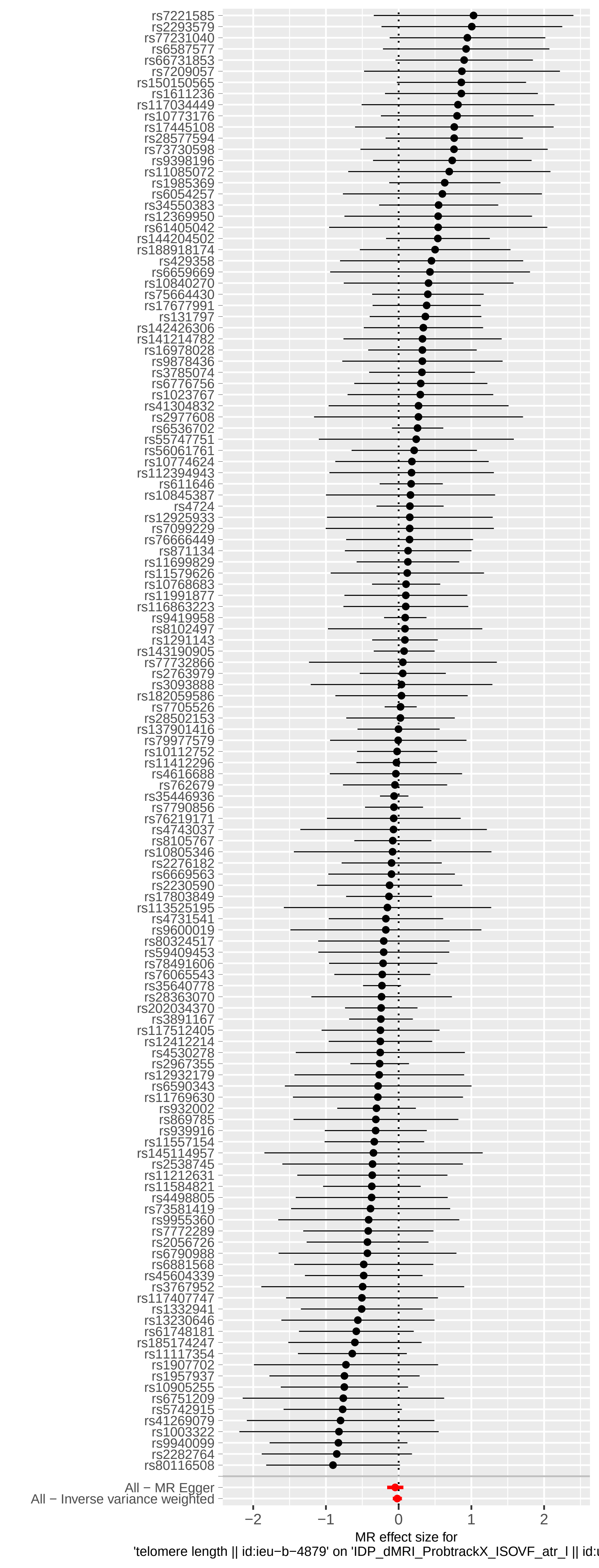
rs7221585
rs2293579
rs77231040
rs6587577
rs66731853
rs7209057
rs150150565
rs1611236
rs117034449
rs10773176
rs17445108
rs28577594
rs73730598
rs9398196
rs11085072
rs1985369
rs6054257
rs34550383
rs12369950
rs61405042
rs144204502
rs188918174
rs429358
rs6659669
rs10840270
rs75664430
rs17677991
rs131797
rs142426306
rs141214782
rs16978028
rs9878436
rs3785074
rs6776756
rs1023767
rs41304832
rs2977608
rs6536702
rs55747751
rs56061761
rs10774624
rs112394943
rs611646
rs10845387
rs4724
rs12925933
rs7099229
rs76666449
rs871134
rs11699829
rs11579626
rs10768683
rs11991877
rs116863223
rs9419958
rs8102497
rs1291143
rs143190905
rs77732866
rs2763979
rs3093888
rs182059586
rs7705526
rs28502153
rs137901416
rs79977579
rs10112752
rs11412296
rs4616688
rs762679
rs35446936
rs7790856
rs76219171
rs4743037
rs8105767
rs10805346
rs2276182
rs6669563
rs2230590
rs17803849
rs113525195
rs4731541
rs9600019
rs80324517
rs59409453
rs78491606
rs76065543
rs35640778
rs28363070
rs202034370
rs3891167
rs117512405
rs12412214
rs4530278
rs2967355
rs12932179
rs6590343
rs11769630
rs932002
rs869785
rs939916
rs11557154
rs145114957
rs2538745
rs11212631
rs11584821
rs4498805
rs73581419
rs9955360
rs7772289
rs2056726
rs6790988
rs6881568
rs45604339
rs3767952
rs117407747
rs1332941
rs13230646
rs61748181
rs185174247
rs11117354
rs1907702
rs1957937
rs10905255
rs6751209
rs5742915
rs41269079
rs1003322
rs9940099
rs2282764
rs80116508
All − MR Egger
All − Inverse variance weighted
−2
−1
0
1
2
MR effect size for
'telomere length || id:ieu−b−4879' on 'IDP_dMRI_ProbtrackX_ISOVF_atr_l || id:u
